# Extraction of Teeth with the Proper Use of Elevators to Conserve the Investing Tissues

**Published:** 1918-07

**Authors:** H. M. Feldman


### EXTRACTION OF TEETH WITH THE PROPER USE OF ELEVATORS TO CONSERVE THE INVESTING TISSUES

BY DR. H. M. FELDMAN

Our earliest conception of the extraction of teeth seems to be closely linked with the custom of the use of the "key" instrument for the elevation of a root from its alveolar bed. As time and man went on, the "old-fashioned" key gave way to the "beak" forceps. The root elevator does not seem to have won much favor in the hands of the present day practitioner, but it is the opinion of the writer, that, with proper instruction and training in their use, no surgical instrument in the dental surgeon's armamentarium would be so much in demand as the root elevator.

It is the custom of the writer to stand in front of his patient and extract all teeth and roots while in this position, just as the general practitioner of surgery stands facing the field of operation. This is the truly surgical attitude, and the really common-sense position to assume, because the surgeon can thus best control the conditions that come up during the course of the operation.

This attitude, then, necessitates the use of the left hand for the manipulation of the instrument when operating on the patient's right side of the mandible, and the reverse for the patient's left side of the face. While the use of the left hand may appear to some as a rather difficult technic it really is quite simple to develop ambidexterity in the use of the root elevators; and the satisfaction which the operator derives from a successful operation in this manner is most gratifying to all concerned.

For the extraction of anterior teeth with their crown portion intact, the use of root elevators, while not absolutely contra-indicated, is not of any material benefit such as would give them preference over beak forceps. But where the crown is so broken down as to necessitate extended subgingival application of force, a root elevator with grooved, concaved beak, continuous with the long axis of the shank of the instrument is a very positive assistant.

### TECHNIC FOR USING ELEVATORS

The instrument is inserted subgingivally and force applied in such manner as to bring the elevator between the root and the alveolus. With careful wrist movement, it will be found that by scarcely disturbing the investing tissues, the root will be extruded from its alveolar bed. In many instances, the root is entirely removed by this means, while in other cases, subsequent to loosening by the elevator, a beak forceps is applied to remove the partially dislocated root.

Many practitioners will readily concede that lower bicuspids are easily fractured at the apical third following the primary resort to the beak forceps. For such extractions, the root elevator is pre-eminently indicated. No epithelial tissue, labially or lingually, is disturbed, no alveolar tissue fractured. Post-extraction pain is practically eliminated and the chances of infection still further reduced.

Lower third molars are most happily removed by the proper manipulation of the root elevator, used, as before, on the principle of lever and fulcrum. No matter how broken-down might be the crown portion, the roots yield quickly to the force of the elevator. In fact, lower molars generally, are removed simply by applying the nose of the elevator between the roots and wedging it in between the roots using one root against the other until it pops one of the roots out with the other materially loosened.

Subgingival removal of upper molar roots is just as easily accomplished by the leverage elevator and all danger of antrum perforation is removed. This is quite an important point to bear in mind, knowing as we do that so often do molar roots extend to or into the maxillary sinus and the application of the beaks of the forceps with an upward push is quite capable of forcing such a root or roots into the sinus.

Root elevators are especially indicated in the extraction of deciduous teeth and roots because their use permits of extreme care in conserving the tissues investing the permanent teeth.

In conclusion, the writer wishes to say, that not the least advantage gained from the use of the elevators is the psychic effect on the patient, who, being familiar only with beak forceps, does not realize very often that the tooth or teeth are being removed when the elevators are applied, thinking that such a step is only preparatory to the application of the forceps. Behold their amazement to find that the roots have sprung out! In the writer's practice, the root elevator has been for some years, and will continue for many more until a better method comes to his attention, to be one of the most essential instruments for the removal of teeth and roots.—*Dental Items of Interest.*

